# Neutrophil Extracellular Traps in *Candida albicans* Infection

**DOI:** 10.3389/fimmu.2022.913028

**Published:** 2022-06-16

**Authors:** Yufei He, Jia Liu, Yutong Chen, Lan Yan, Jianhua Wu

**Affiliations:** ^1^ Department of Dermatology, Changhai Hospital, Second Military Medical University (Naval Medical University), Shanghai, China; ^2^ School of Pharmacy, Second Military Medical University (Naval Medical University), Shanghai, China

**Keywords:** neutrophils, neutrophil extracellular traps, *Candida albicans*, virulence factors, innate immunity

## Abstract

*Candida albicans* is the most common pathogen causing clinical *Candida* infections. Neutrophils are a key member of the host innate immunity that plays an essential role in clearing invading *C. albicans*. In addition to the well-known defensive approaches such as phagocytosis, degranulation, and reactive oxygen species production, the formation of neutrophil extracellular traps (NETs) has also become an important way for neutrophils to defend against various pathogens. *C. albicans* has been reported to be capable of activating neutrophils to release NETs that subsequently kill fungi. The induction of NETs is affected by both the morphology and virulence factors of *C. albicans*, which also develops specific strategies to respond to the attack by NETs. Our review specifically focuses on the mechanisms by which *C. albicans* triggers NET formation and their subsequent interactions, which might provide meaningful insight into the innate immunity against *C. albicans* infection.

## Introduction

As the most prevalent opportunistic fungi in humans, *Candida* spp. frequently cause local infection by invading the host mucosa and tissues and, occasionally, lead to life-threatening invasive candidosis. Recently, the widespread use of broad-spectrum antibiotics, glucocorticoids, and immunosuppressants, and the continuous development of medical treatments such as tumor chemotherapy and organ transplantation, has increased the yearly incidence of invasive candidosis. Furthermore, *Candida albicans* is the pathogen that accounts for the highest proportion of infections (1–4). *C. albicans* possesses strong morphological plasticity and can grow in the form of yeast, pseudohyphae (rarely seen), or hyphae (5), which is closely related to its pathogenicity (6, 7). For instance, the morphological transition from yeast to hyphae is regarded as one of the significant virulence factors of this microbe.

Innate immunity is the first line of defense of the host against pathogens and its vital role in clearing *C. albicans* infection requires the activity of immune cells such as neutrophils, macrophages, dendritic cells, and natural killer cells. Among them, neutrophils are especially indispensable that they can kill *C. albicans* through phagocytosis, degranulation, the production of reactive oxygen species (ROS), and the formation of neutrophil extracellular traps (NETs) (8). The yeast form of *C. albicans* can be phagocytosed by neutrophils, thereby initiating a series of efficient killing mechanisms (9). In contrast, hyphae are too large to be engulfed and, therefore, the formation of NETs provides a viable alternative extracellular pathway for neutrophils to kill them (10).

## Brief Overview of NET Formation

NETs, large extracellular networks consisting of DNA fibers 15–17 nm in diameter and spherical proteins approximately 25 nm in diameter, are mainly released by neutrophils to defend against the invasion of various pathogens (11, 12). These protein constituents are histones, neutrophil elastase (NE), myeloperoxidase (MPO), defensin, calprotectin, and actin (12, 13). According to the different mechanisms of NET formation, the DNA may come from either nucleus or mitochondria.

Originally, NETs were thought to be released through a cell death process that was later named “NETosis” (14). First, NE leaks out of the granular vesicles and translocates to the nucleus to degrade histones, leading to chromatin decondensation. The nuclear membrane disintegrates, releasing the DNA outside to mix with granular proteins in the cytoplasm, and these components are finally released together when the plasma membrane ruptures. This process is ROS-dependent and usually takes 3 to 4 hours. Stimuli such as phorbol 12-myristate 13-acetate (PMA), bacteria, and fungi enable neutrophils to assemble a large protein complex, nicotinamide adenine dinucleotide phosphate (NADPH) oxidase or phagocyte oxidase (Phox) (15). ROS generated after oxidase activation is the initiating factor in the release of MPO and NE from the granules. However, Pilsczek et al. (16) found that neutrophils released NETs 10 minutes after exposure to *Staphylococcus aureus*.

Both nuclear DNA and granular proteins are expelled in a vesicle-wrapped form, and eventually assembled into NETs in the extracellular space. This rapid release of NETs, which is not accompanied by cell death or ROS-dependent, is considered an antimicrobial behavior of neutrophils in the early stage of infection. Studies suggests that ROS-independent NET formation could be initiated by protein-arginine deiminase 4 (PAD4)-mediated histone citrullination (17). Yousefi et al. (18) reported another formation mechanism that does not involve cell death, in which mitochondrial DNA is rapidly released in 15 minutes with the requirement of ROS. Previously, a similar mechanism was described for the antibacterial action of eosinophils that also release NETs (19).

## Different Forms of *C. albicans* Induces Neutrophils to Release NETs

Multiple stimuli activate neutrophils to release NETs (20–22), which following the initial discovery in 2004, were induced by interleukin (IL)-8, PMA, and lipopolysaccharide (LPS) (11). Approximately 2 years later, Constantin et al. (10) reported that *C. albicans* also triggered the formation of NETs. Subsequently, studies were conducted to explore the correlation between *C. albicans* infection and NET formation, including those indicating that the morphology of *C. albicans* affected its stimulation of the release of NETs by neutrophils.

**Figure 1:**
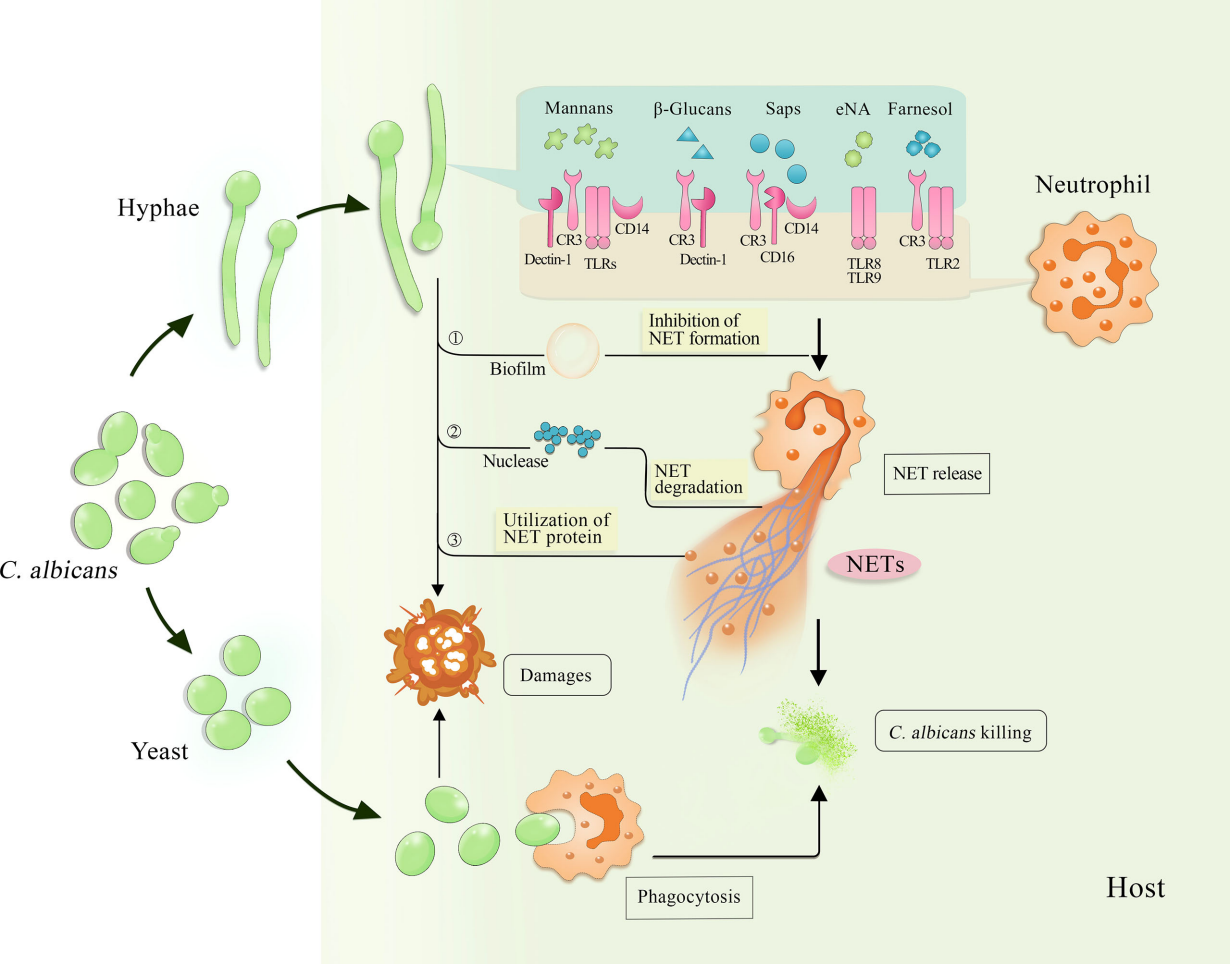
Interaction among *C. albicans*, neutrophils and NETs. The invading *C. albicans* can be cleared by neutrophils through different killing approaches, such as phagocytosis targeted to yeast and NET release induced by hyphae. Several stimuli derived from *C. albicans* hyphae, including mannans, β-glucans, Saps, eNA and farnesol, activate various receptors on the surface of neutrophils and trigger NET release. Consequently, NETs capture and kill both yeast and hyphae. Furthermore, *C. albicans* inhibits NET release by forming biofilms and degrades NETs by secreting nuclease, while using NET proteins to strengthen their invasiveness. NETs, neutrophil extracellular traps; Saps, secreted aspartic proteases; eNA, extracellular nucleic acids; CR3, complement receptor 3; TLRs, toll-like receptors.

Neutrophils sense the size of the pathogens to selectively release NETs in response to large ones such as *C. albicans* hyphae (23) (**Figure 1**). With microbes small enough to be engulfed, dectin-1-mediated phagocytosis downregulates the translocation of NE into the nucleus to inhibit NETosis, thereby avoiding unnecessary histopathological damages. The selective release was not observed in neutrophils of dectin-1-deficient mice because of the genetic disruption of phagocytic function, which means that pathogens in all sizes could induce NETs. As for hyphae, since they cannot be phagocytosed, there is no similar inhibition on NETosis.

Contrary to the widely accepted perspective that hyphae induce NET formation, whether yeast can activate neutrophils to release NETs is still controversial. In an experiment by Kenno et al. (24) where human neutrophils and *C. albicans* were co-incubated, both yeast and hyphae were shown to induce the early release of NETs. In addition, 4 hours later, the yeast could not continuously induce NET formation, whereas the hyphae still could. After a 4-hour co-incubation of neutrophils and heat-inactivated yeast, a certain number of NETs were observed. Ermert et al. (25) used neutrophils from mice of different strains and obtained similar results, indicating that similar to hyphae, yeast could also induce NET formation although it was significantly weaker. However, a yeast-locked *C. albicans* mutant *hcg1* failed to trigger NET release by neutrophils (23), which raised questions about the NET-inducing ability of yeast. Considering that the co-incubation environment (such as the 37°C temperature) induces filamentation of yeast, various measures have been taken to sustain the yeast form in the experiments above. In view of the obviously inconsistent results, a more comprehensive and rigorous experiment should be designed to determine whether NETs can be induced by yeast.

## Multiple Virulence Factors of *C. albicans* Play a Role in NET Induction

Several factors contribute to virulence in *C. albicans*, such as cell wall polysaccharides, surface proteins, and proteases that are critical to the occurrence of the diseases and activation of the host immune responses. To define the roles they play in inducing the formation of NETs, Zawrotniak et al. (26) isolated and purified a variety of virulence factors from *C. albicans* hyphae. After cultivation with neutrophils for 3 hours, the main constituents of the cell wall such as mannans, β-glucans, and proteins including secreted aspartic proteases (Saps) and surface proteins were observed to induce NET release (26) (**Figure 1**). The induction intensity of mannans exceeded that of PMA, which is usually used as a potent NET inducer.

CD11b/CD18, CD14, dectin-1, and toll-like receptors (TLRs) are the major receptors on the surface of neutrophils that recognize mannans (26–28). This is of great importance to the host, because mannans locate in the outermost layer of the fungal cell wall and cover other constituents such as β-glucans. Consequently, mannans can easily prevent these constituents from being recognized by the host cells, thereby resulting in a decline of the immune responses (29, 30). After recognition, mannans trigger NET formation through both ROS-dependent and -independent pathways. Moreover, Saps induce NETs to different degrees primarily by interacting with CD11b/CD18, CD16, and CD14 receptors, including Sap4 and Sap6 that exhibit relatively efficient NET-inducing abilities (26).

β-glucans were the first substances discovered to induce NET formation, but the signaling pathways involved have not been clearly elucidated. Previous work demonstrated that β-glucans triggered NET formation in a concentration-dependent manner, primarily through an ROS-dependent pathway (26, 31). However, Byrd et al. (32, 33) suggested the opposite that β-glucan-induced NET release required not ROS but the extracellular matrix (ECM), in which β-glucan in the context of fibronectin (FN) had been confirmed to mediate rapid (in 30 minutes) homogeneous aggregation and NET formation.

Although the importance of the recognition of β-glucan particles by dectin-1 has already been emphasized (34–36), in a study that focused on the effect of neutrophils on *Saccharomyces cerevisiae* and its β-glucan-containing capsular constituent zymosan, Bruggen et al. (37) found that complement receptor 3 (CR3, also known as αMβ2 or macrophage antigen [Mac]-1; consisting of CD11b/CD18), rather than dectin-1, was responsible for β-glucan recognition. This finding was supported by data from experiments with neutrophils of a patient diagnosed with leukocyte adhesion deficiency type 1 (LAD-1) syndrome who had defects in CD11b/CD18 expression. Subsequently, Byrd et al. (32) similarly stressed that CR3 is the main receptor on human neutrophils that recognizes β-glucans. However, another study presented the challenge that the induction of NET formation by β-glucans was ROS-dependent and required dectin-1 (31). The activation of CR3 was indirect and likely the result of β-glucan recognition by dectin-1 (36). The current view is that both dectin-1 and CR3 participate in the formation of β-glucan-triggered NETs (26). Further studies are needed to explicitly clarify the interrelationship between β-glucans and diverse pattern recognition receptors (PRRs) as well as the subsequent reactions.

Interestingly, CR3 appears to be persistently present, though the stimuli and activated pathways vary. As a member of the β2 integrin family, CR3 initiates a complex integrin cross-talk in response to *C. albicans* hyphae or β-glucan and FN, contributing to differential expression of β1 integrin VLA3 (or α3β1) and VLA5 (or α5β1) that regulate homotypic aggregation and NET formation respectively (38). CR3 has two spatially distinctive binding sites, lectin-like domain and I-domain (39, 40). The lectin-like domain tends to be occupied by certain pathogen-associated molecular patterns (PAMPs), which makes CR3 a PRR for fungal surface molecules such as β-glucans (41, 42). The I-domain is a highly farraginous binding site that can bind more than 30 ligands with completely different structures, including intercellular cell adhesion molecule-1 (ICAM-1), heparan sulfate (HS), and FN mentioned above (43). Although no evidence supports the notion that CR3 is involved in the positive effect of FN on β-glucan-triggered NET formation, this hypothesis does deserve attention and further investigation. Considering that ICAM-1 and HS are both associated with NET release by neutrophils (44–47), it is reasonable to speculate that CR3 plays a central role in NET formation.

Biofilm formation is another virulence factor of *C. albicans* and its covering of the surface provides the fungus with the best local environment for growth in the host. Furthermore, this covering constitutes the first contact of biofilms with host immune cells and the subsequent interaction. When evaluating the effect of extracellular nucleic acid release in *C. albicans* biofilm formation on NETs, Smolarz et al. (48) found that fungal nucleic acids triggered NET release (**Figure 1**). Extracellular nucleic acids purified from mature biofilms or nucleic acids isolated from intact fungal cells have the potential to activate neutrophils to release NETs *in vitro*. These DNA and RNA molecules trigger NET formation through the ROS-dependent pathway, in which TLR8 and TLR9 are involved (48, 49). As biofilms form, information is transmitted between *C. albicans* cells through a quorum sensing (QS) mechanism (50, 51) facilitated by QS molecules (QSMs) mainly including farnesoic acid, tyrosol, and farnesol. Zawrotniak et al. (52) demonstrated that farnesol induced ROS-dependent NET formation through the recognition by CR3 and TLR2 (**Figure 1**), whereas farnesoic acid and tyrosol did not. However, recent studies suggest that biofilms inhibit the formation of NETs (53, 54).

The total amount of NETs induced by fungus with biofilms is significantly lower than that by planktonic *C. albicans*. Once biofilms are destroyed, NETs will increase. The inhibition caused by biofilms might be attributed to the obstruction of ROS production, which probably involves ECM because disrupting it impairs the inhibitory effect (55). In addition, Hoyer et al. (56) reported that *C. albicans* biofilms treated with appropriate concentrations of echinocandin unexpectedly stimulated the formation of NETs. In summary, although some elements of biofilms were found to trigger NET formation, the inhibitory action exhibited by biofilms as a whole has been confirmed.

## Interaction Between NETs and *C. albicans*

In 2006, NETs were first reported to capture and kill both *C. albicans* yeast and hyphae (10) (**Figure 1**), but their antifungal mechanisms are unknown. Generally, the dense reticular structures act as a physical barrier to prevent the dissemination of trapped pathogens in the host. Further studies showed that MPO- or NADPH oxidase-deficient mice releasing insufficient amounts of NETs (57) eventually died from invasive candidosis, whereas MPO-deficient mice survived infections caused by yeast-locked mutants (23). These findings confirmed the crucial role of NETs in controlling hyphae growth and the capability of neutrophils with impaired NET release to clear the yeast form of *C. albicans* through phagocytosis. Furthermore, studies including mouse neutrophils in the mucosal candidosis model established with transparent zebrafish swim bladders, showed a decrease in hyphal damage caused by neutrophil deficiency and insufficient release of NETs (58). Neutrophils of patients experiencing chronic granulomatous disease (CGD) with impaired ROS production had difficulty in controlling hyphal growth because of delayed NETosis (59). The findings of all these studies suggest that the formation of NETs is an indispensable strategy for neutrophils to fight the invasion of *C. albicans* hyphae.

The main constituents of NETs are proteins and DNA framework and, consequently, these proteins and their corresponding functions have attracted the attention of researchers dedicated to elucidating the antimicrobial mechanisms of NETs. Urban et al. (60) used proteomics approaches to identify 24 NET proteins, some of which also exist in the cytoplasm of unstimulated neutrophils. Among them, a heterogeneous dimer calprotectin was found to be essential in the clearance of *C. albicans* infection, although a subsequent study suggested that the proteins contained in NETs varied because of diverse stimuli (61). NETs with a lack of calprotectin completely lose their antifungal activity *in vitro* (60). As a divalent metal-ion chelate, calprotectin exerts antifungal activity by depleting Zn^2+^, Mn^2+^, or both, which are required for microbial proliferation (62, 63). Moreover, histones or histone-like peptides have been found to kill *Cryptococcus neoformans* and *Candida tropicalis*, but not *C. albicans* (10). Other proteins like MPO and defensins are potent antimicrobials (57, 64), but their specific roles in NETs-mediated fungal clearance have not yet been elucidated.

Although the antifungal activity of NETs has been shown in numerous studies, Menegazzi et al. (65) argued that NETs captured microbes but could not kill them. After incubation with NETs induced by PMA pretreatment for 30 minutes, the captured *C. albicans* spores (or *S. aureus*) were released with the help of DNase disrupting the framework of NETs, and then recovered. Nevertheless, the following two points should be considered. 1) If the co-incubation time is extended, would the trapped pathogens still survive, and 2) since *C. albicans* spores and *S. aureus* can be phagocytosed by neutrophils, would the selection of filamentous fungi as alternative microbes in the experiment produce the same result?

*C. albicans* develops several defensive strategies to deal with NETs killing (**Figure 1**), such as biofilm formation that inhibits the release of NETs (already reviewed above). In addition, *C. albicans* secretes DNase or 3′-nucleotidase/nuclease to disintegrate the NETs structure, thereby escaping from the traps (66, 67). Nevertheless, degradation of DNA cannot essentially protect the fungus from the progressing damage caused by multiple antifungal proteins of NETs. Unexpectedly, *C. albicans*, in turn, takes advantages of these proteins to enhance its colonization and tissue damages to the host. NET-related proteins such as NE bind to adhesin Als3 of the lectin-like sequence protein family as well as other atypical proteins exposed on the surface of *C. albicans* cells (68). *C. albicans* cells attached by the mixture of NET proteins are significantly more destructive to human epithelial cells than the fungi themselves are. More details about the interaction between NETs and *C. albicans*, the hyphal form in particular, remain to be further elucidated.

## Conclusion

It is universally acknowledged that neutrophils are of great importance in infectious diseases. Over the past two decades, the formation of NETs and their function as a new antimicrobial method have attracted considerable attention, though dysregulated NETs can cause tissue damages and immune-related diseases (12). To date, *C. albicans* hyphae have been confirmed to be capable of activating neutrophils to release NETs, which involves ROS-dependent and -independent pathways, whereas the NET-inducing ability of yeast remains controversial. The key point of this distinction is that the phagocytosis by neutrophils is not targeted to hyphae but to yeast, which might hinder the formation of NETs. Studies have shown that a variety of virulence factors of *C. albicans*, such as mannans, β-glucans, and Saps are efficient stimuli to induce NET release. CR3 appears to be the central receptor in this process, although additional evidence is needed. Extracellular nucleic acids and farnesol have similar actions, but biofilms exhibit an inhibitory effect on NET formation. Following their release, NETs begin to capture pathogens. Although some researchers are of the opinion that captured microbes will survive inside NETs, most are inclined to believe that NETs will kill pathogens through the actions of their antimicrobial proteins, especially calprotectin. Since filaments are too large to be phagocytosed by neutrophils, NETs are vital to clearing *C. albicans* hyphae. Furthermore, *C. albicans* also attempt to evade destruction by NETs through the secretion of nuclease and formation of biofilms, while using NET proteins to strengthen their invasiveness.

Currently, the intricacies of the interplay between *C. albicans* and NETs released by neutrophils is gradually being elucidated, but the precise mechanism underlying *C. albicans*-induced NET formation and regulation have not been reasonably clear. A recent study suggested that dectin-2 recognizes *C. albicans*, thereby triggering ROS-independent NET formation, which requires PAD4-mediated histone citrullination (69). However, another study demonstrated that PAD4 was not involved in the NET formation induced by *C. albicans* (70). In view of the limited relevant research findings, it is difficult to draw a definitive conclusion.

Several fundamental scientific questions remain to be answered, such as: 1) whether *C. albicans* yeast can induce NET formation, 2) whether CR3 on the surface of neutrophils always participates in NET formation triggered by various stimuli, 3) the signaling pathways and regulatory factors, and 4) the mechanisms underlying the actions of antifungal proteins in NETs. Therefore, more in-depth researches are urgently needed. Nevertheless, we hope that this review provides meaningful insight into the study of the innate immunity driving the clearance of *C. albicans* infection, which might eventually contribute to the developing treatment strategies in clinical practice.

## Author Contributions

YH: Conceptualization (equal), Writing—original draft preparation (lead); JL: Investigation (equal), Visualization (equal); YC: Investigation (equal), Visualization (equal); LY: Funding acquisition (lead), Writing—review and editing (equal); JW: Conceptualization (equal), Writing—review and editing (equal). All authors contributed to the article and approved the submitted version.

## Funding

This work was supported by National Natural Science Foundation of China (82173867), Shanghai Science and Technology Innovation Action Plan, International Science and Technology Cooperation Project (21430713000), Shanghai Science and Technology Support Project in the Field of Biomedicine Project (19431901300), Shanghai Pujiang Program (21PJD0081).

## Conflict of Interest

The authors declare that the research was conducted in the absence of any commercial or financial relationships that could be construed as a potential conflict of interest.

## Publisher’s Note

All claims expressed in this article are solely those of the authors and do not necessarily represent those of their affiliated organizations, or those of the publisher, the editors and the reviewers. Any product that may be evaluated in this article, or claim that may be made by its manufacturer, is not guaranteed or endorsed by the publisher.
